# Correction of Deep Overbite by Using a Modified Nance Appliance in an Adult Class II Division 2 Patient with Dehiscence Defect

**DOI:** 10.1155/2018/9563875

**Published:** 2018-09-06

**Authors:** Zhujun Li, Zhengxi Chen, Jian Sun, Li'an Yang, Zhenqi Chen

**Affiliations:** ^1^Department of Orthodontics, Shanghai Ninth People's Hospital, School of Stomatology, Shanghai Key Laboratory of Stomatology, Shanghai Jiao Tong University, Shanghai, China; ^2^Department of Stomatology, Shanghai East Hospital, Tongji University School of Medicine, Shanghai, China; ^3^Department of Stomatology, Xin Hua Hospital Affiliated to Shanghai Jiao Tong University School of Medicine, Shanghai Jiao Tong University, Shanghai, China

## Abstract

A modified Nance Appliance (MNA) is introduced as a treatment option for an adult class II division 2 malocclusion (CII/2) patient with deep overbite and dehiscence on the facial root surface of retroclined upper incisors through the cone-beam computed tomography (CBCT). Indications for this modified MNA as well as a brief description of fabrication procedure and biomechanical analysis of the treatment effects are shown in detail. Root control and absolute intrusion without enlarging the bony defect were achieved. The treatment results were satisfying and favorable.

## 1. Introduction

The management of alveolar defects, namely, dehiscence and fenestration in orthodontic patients, remains a tricky task for orthodontists to accomplish. Dehiscence could be presented with the lack of the facial or lingual cortical plate, which has a tendency of exposing the cervical root surface and affecting the marginal bone [1]. Bone dehiscences are common in the mandibular symphysis region before orthodontic treatment, especially among adults [2]. To avoid further risks, the alveolar morphology should be determined before orthodontic treatment. Currently, cone-beam computed tomography (CBCT) is an ideal option chosen in such a clinical dental situation for its ability to provide accurate images of the entire bone structure and visualizing these defects three dimensionally [3, 4]. According to the research of Fuhrmann [5], 80% of defects identifiable on CT scan images were not readily visible on the lateral cephalograms.

Clinically, patients with characteristic angle class II division 2 malocclusion (CII/2) always have manifestations including a prognathic maxilla, retroclined maxillary central incisors with alveolar bone defect on the cervical root surface, a retrusive mandible, upright mandibular incisors, a deep overbite, and a deep curve of Spee. The updated study suggested that early intervention should be taken to intercept, disrupt, and diminish the effects of malocclusions [6]. Coskuner and Ciger [7] indicated that in patients with class II/2 malocclusion, the elimination of maxillary interferences may lead to a greater increase in the mandibular dimensions. After eliminating the factors restricting mandibular movement in the transverse and sagittal planes, changes in the mandible and temporomandibular joints were observed. Patients in the pubertal growth period determined using the cervical vertebral maturation method who exhibited class II division 2 malocclusion deficiency were considered eligible for early intervention [8, 9]. For adult class II/2 patients who have no growth potential, a possible dental compensation or surgical-orthodontic treatment might be considered a favorable choice; many patients were still expecting an invasive method of treatment. According to the literature research, the class II group has a greater prevalence of dehiscences and fenestration than the class I and class III groups. Fenestrations has greater prevalence in the maxilla, but more dehiscences were found in the mandible [10]. Although the limitation of alveolar bone modeling and remodeling during intrusion of maxillary incisors existed [2, 5] and the alveolar bone dehiscence might get worse after treatment [11], good alveolar bone adaptation could still be achieved with care. Decker and Chen [12] demonstrated good upper alveolar bone adaptation after 32 years of follow-up by a case report.

In this case, we describe our treatment of an adult male patient diagnosed as class II/2 malocclusion with undersized lateral incisors and a dehiscence defect through the bone extending from the buccal root surface of upper retroclined incisors using a modified Nance Appliance (MNA).

## 2. Diagnosis and Etiology

A 27 years and two months old male patient who had a chief complaint of upper front teeth lingually tipped came to our department of orthodontics for further treatment. He pointed out that his mother shared the similar malocclusion.

The clinical examination showed a mild convex profile, a decreased anterior lower facial height, a prominent chin button, and a symmetrical face in frontal view (Figure 1). No significant temporomandibular joint discomfort was found. He was healthy, with no specific medical problems. Intraoral examination and dental casts showed that the patient was in the permanent dentition. He had an overjet of 3 mm, an overbite of 6 mm, retroinclined upper incisors, two undersized upper lateral incisors, and mild crowding in both dental arches. Class II canine and molar relationship were shown on the right side while class I canine and molar relationship was shown on the left side. Clinical periodontal examinations were evaluated at 6 sites on the number of teeth, which showed the means of probing depth (PD: 2.7 mm), gingival index score (GI: 1.5), and bleeding on probing (BOP) positive percentage (16.7%). In addition, his oral hygiene was unsatisfactory and needed periodontal scaling before fixed orthodontic treatment (Figures 1 and 2).

The panoramic radiograph showed missing third molars. The condyles appeared normal in size and form. Normal root length and bone height were present, with no caries or other pathology noted (Figure 3). Dehiscence was found on the facial root surface of upper central incisor through the CBCT (Figure 3). The lateral cephalometric analysis indicated a skeletal class II pattern (ANB, 5.2°; wits appraisal, −1.4 mm) with a decreased lower anterior facial height (FMA, 23.7°; face height ratio, 51.7%). The maxillary incisors and the mandibular incisors were retroclined (U1-SN, 76.8°; IMPA, 79.7°). As a result, the interincisal angle was increased (U1/L1, 172.7°) (Figure 3 and Table 1). Cephalogram suggested that he had a class II skeletal pattern, hypodivergent growth, and decreased lower anterior facial height.

According to the examination above, the patient was diagnosed of CII/2 subdivision malocclusion with retroinclined upper incisors, undersized lateral incisors, dehiscence defect in the maxillary anterior alveolar bone, and deep overbite.

## 3. Treatment Objectives

The treatment objectives were to establish ideal overbite, overjet, and class I molar relationship on both sides, obtain favorable inclination of the maxillary and mandibular incisors, correct the maxillary and mandibular dental midline, relieve crowding, align the lower arch, provide proper space for the prosthesis of the undersized lateral incisor, maintain the profile, and avoid excessive lip protrusion, further dehiscence defect, and gingival recession as well.

## 4. Treatment Alternatives

The following two treatment alternatives were considered.

- Option 1: the first one is just to rectify the inclination of the incisors and relieve crowding of the teeth. The canine and molar relationships would retain class II on the right side. Also, the denture midline would not be corrected either
- Option 2: the second one is to regain the space for dental prosthesis of undersized lateral incisors, to correct the canine and molar relationship, and to adjust maxillary denture midline. The maxillary molars would be distalized by miniscrew implant anchorage to relieve the crowding

After full communication, he rejected the second option and emphasized that his chief concern was to correct retroclined upper incisors. He wanted to maintain the occlusion of the posterior element. According to this chief complaint, we reached a consensus and chose the first option. However, root torque control during tooth treatment is a tricky task for many orthodontists to accomplish. In this case, if space created by molar distalization is not enough for relieving crowding and controlling the movement of roots of upper incisors, the upper incisors will inevitably be flared at the initial alignment stage which will result in a worsening of the alveolar bone defect. To avoid this problem, we fabricated the MNA, which will be described below in detail.

## 5. Treatment Progress

The treatment was commenced with the placement of the MNA. The appliance consisting of two connecting palatal archwires made of 1.2 mm stainless steel wire, palatal button made of acrylic resin, and two additional hooks made of 0.7 mm stainless steel wire was cemented on both upper first molars to keep the crown of the upper incisors in position (Figure 4). To effectively control the movement of upper incisors, two metal buttons were placed gingivally on the lingual surface of upper incisors and were activated by stainless steel ligature tying to the hooks on MNA.

Self-ligating brackets (Damon Q, 0.022 × 0.025, High Torque, ORMCO) were bonded in the upper arch with a 0.013-inch CuNiTi wire engaged. Additionally, the brackets were placed 0.5 mm close to the incisal edge of upper incisors for the intrusion. Meanwhile, glass ionomer cement was cemented on the occlusal surface of the upper first molars as an occlusal stop to eliminate occlusal inference (Figure 5). The initial aligning and leveling stage took place over the first 3 months while the roots of upper incisor were moving lingually.

At month 4, the upper arch form was changed from square to ovoid. The MNA and occlusal stop were removed. After the removal, the cephalometric radiograph was retaken for observing the roots of upper incisors. As was shown in Figure 6, in the upper arch, the movement of the crown of upper incisors was under control as well as the root retraction without the exaggerated dehiscence on the buccal root surface of upper incisor.

For further alignment and leveling, a 0.014 × 0.025-inch CuNiTi archwire was changed by a 0.017 × 0.025-inch NiTi archwire. At month 12, the deep bite was almost corrected. At this point, the lower arch was bonded and leveled (Figure 7). To achieve more favorable torque expression in the upper teeth, 0.019 × 0.025-inch stainless steel archwires were engaged in the end (Figure 8). The near end stage of treatment was to adjust midline and occlusal relationship for better intercuspation. The patient was instructed to wear class II elastics (5/16, 3.5 orz) full time for three months. In the end, the teeth were well aligned with the good intercuspal relationship, and the functional class II canine and molar relationship on the right side was achieved. After 22 months, the appliance was removed, the upper and lower Hawley retainers were placed with a protocol of wearing full time for the first six months.

## 6. Treatment Results

Patient cooperation throughout the treatment was excellent. The posttreatment records indicated that the treatment objectives were achieved. The facial photographs demonstrated significant improvements in his soft tissue profile with increased anterior lower facial height. He was very happy with the outcome (Figure 9).

Intraorally, the retroclination of upper and lower incisors was corrected. Optimal overjet (4 mm) and overbite (2.0 mm), a class I canine and molar relationships on the left side, and a class II canine and molar relationship on the right side were reached (Figures 9 and 10). Remarkably, the torque of the upper incisors was in good control as expected (U1-SN, 102.4°). Root control and absolute intrusion of the maxillary incisors were achieved at the initial stage. The lingual movement of root of upper incisors was attained easily by using a high-torque version of self-ligating brackets. Meanwhile, the crown was avoiding extra labial tipping all through the treatment (Figure 11 and Table 1). Through good oral health education, the patient became more conscious of oral hygiene after the treatment. Clinical indicators including PD (2 mm), GI (1), and BOP positive percentage (0%) show a healthy periodontal state after treatment. Therefore, the absolute intrusion of the maxillary incisors did not worsen the periodontal problems but enhance the bony support for teeth.

The panoramic radiograph showed no significant bone loss or root resorption, and all tooth roots were parallel to each other (Figure 11). Cephalometric superimposition registered on the SN line showed the intrusion of the maxillary incisors by 2 mm with lingual root movement by 25.6 degrees and the increased mandibular plane angle (FMA,25.9°) (Figure 12 and Table 1). As a result, the anterior lower facial height was increased to 2 mm. According to CBCT image, we had found excitedly the original dehiscence on cervical root surface of upper incisor restored on the labial surface instead of getting worse (Figure 11). There was a thin layer of bone covering on the cervical root surface of upper incisor visibly.

After 22 months of treatment, the patient was satisfied with the outcome. However, the patient studied abroad later and out of contact. We lost follow-up unfortunately.

## 7. Discussion

As to CII/2 malocclusion, the objectives of treatment mainly focus on correction of anterior deepbite, correction of upper incisor inclination, and correction of class II molar relationships [13–16]. Clinically, correction of deep overbite can be achieved by molar extrusion, incisor intrusion, or a combination of these two types of tooth movement. Extrusion of posterior teeth is stable in growing patients when the growth potential of mandibular condylar remains. In adult patients, posterior teeth extrusion is probably counteracted by the posterior occlusion, especially in the hypodivergent skeletal pattern.

For this specific patient, to some extent, deep overbite could be corrected by intruding the maxillary and mandible incisors. Nevertheless, with a dehiscence defect existed in maxillary anterior teeth, a careful consideration of avoiding further dehiscence of the alveolar bone should be addressed. According to Carranza [17], dehiscences were defined as bony defects in which the denuded areas involve the alveolar bone margin. Previous studies used various criteria for the definition of dehiscences, including any defect greater than 1 mm near the cementoenamel junction (CEJ) [18], 4 mm apical to the interproximal bone crest [19–21], and exposure of half of the root [22]. The presence of these buccal alveolar bone defects weakened the bony support for teeth. Inadequate bone support during orthodontic tooth movement may have deleterious effects on teeth and the periodontium [23]. Additionally, rapid movement of these teeth may also reduce the amount of radicular bone, enlarge the alveolar defect, and increase the risk of gingival recession. Studies have shown greater prevalence of alveolar defects on the buccal surface than on the lingual surface [1, 21]. This may be attributed to narrow bone at the buccal surface, where the amount of marrow bone is less dense than that in the lingual region.

Currently, periodontally accelerated osteogenic orthodontics (PAOO) is one of the most classic techniques for the alveolar bone defect. This approach includes buccal and lingual full-thickness flaps, selective partial decortication of the cortical plates, concomitant bone grafting or augmentation, and primary flap closure [24]. The aim of the surgery is focused on covering bony dehiscences and reshaping alveolar bone, thus provides additional support for the roots of the teeth and diminishes risk of periodontal recession. However, for this patient, PAOO is not a smart choice. Patients with characteristic CII/2 malocclusion always show a prognathic maxilla, retroclined maxillary central incisors, a retrusive mandible, and a deep curve of Spee [6]. Abovementioned PAOO would augment the thickness of alveolar bone and make A point move forward, which would lead to further increase of the prominence of upper lip and an adverse influence on the soft-tissue profile. Therefore, in such cases, a noninvasive and cost-effective method of treatment is needed. Through the CBCT, dehiscence was found on the facial root surface of upper incisor. To avoid enlarged alveolar defect, labial tipping should be under precise control. For this reason, we fabricated the MNA. The two metal buttons bonded gingivally on the lingual surface functioned as the pivot. The labio-lingual inclination of the upper central incisors would move around the pivot which attached to hooks on the MNA. After 0.013-inch CuNiTi archwire was fully engaged into the bracket slots of the malpositioned teeth, the gentle force delivered by the archwire progressively aligned the maxillary teeth. During the initial alignment stage, the crowns of maxillary incisors were held by the hooks and the roots moved lingually. By using the MNA, full control of the tipping of the upper incisors and absolute intrusion of the maxillary incisors were achieved. To avoid deteriorating alveolar bone defect, the gentle force and slow movement of teeth should be taken into account at the initial stage. At the end of treatment, we could find a thin layer of bone covering the cervical root surface of maxillary incisor followed by the successful lingual movement of roots of maxillary incisors.

As known, the interincisal angle has been considered as a factor related to long-term stability. Riedel [25] indicated that a large interincisal angle at the end of treatment would be associated with the relapse of deep overbite. Proper palatal root torque of the maxillary incisors plays an important role in maintaining a normal interincisal angle and establishing anterior occlusal stop [26]. In this case, we corrected the torque of the retroclined upper incisors (Figure 12 and Table 1), which was beneficial to maintain an optimal interincisal angle and overbite stability. The idea that bone traces tooth movement indicates whenever orthodontic tooth movement occurs, the bone around the alveolar socket would remodel to the same extent [27]. When the angle of upper incisors to SN plane changed from 76.8° to102.4°, the anterior alveolar bone underwent remodeling at the same time. The A point moved backward subsequently which caused SNA angle changed from 84.2° to 81.7° (Table 1).

Although, there are some other clinical treatment approaches reported for controlling root torque, such as auxiliary arch, rectangular loops, and reverse-curve archwire. However, the MNA fabricated in this case shows some merits. It could achieve root control and absolute intrusion of the maxillary incisors at the initial stage. Combined with a high-torque version of self-ligating brackets, the lingual movement of root of upper incisors could be attained easily. Remarkably, it is noninvasive, cost-effective, and easy to handle without repeated adjustment.

The patient in this case chose to maintain the dental midline and the occlusion of the posterior element and rejected to restore his undersized lateral incisors. Given the reason that satisfaction with the outcome depends on the expectations of patients, their point view of the assessment of final aesthetics is decisive. Therefore, acceptable overjet and a class II canine and molar relationship on the right side were achieved after treatment. The posttreatment occlusion was improved both functionally and aesthetically. The outcome of treatment was favorable. Further follow-up on the stability of the MNA would be scheduled.

## 8. Conclusions

The clinical management for orthodontic patients with alveolar defects as dehiscence and fenestration remains a tricky task for orthodontists to accomplish, especially in adult class II division 2 malocclusion (CII/2) patients with deep overbite and dehiscence on the facial root surface of retroclined upper incisors. The MNA presented in this case showed a favorable potential in achieving satisfying results during active orthodontic treatment.

## Figures and Tables

**Figure 1:**
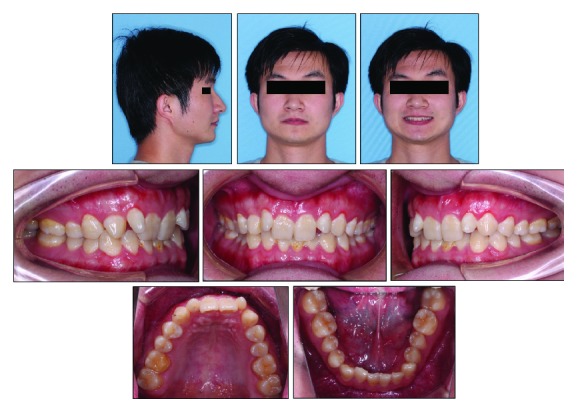
Pretreatment facial and intraoral photographs.

**Figure 2:**
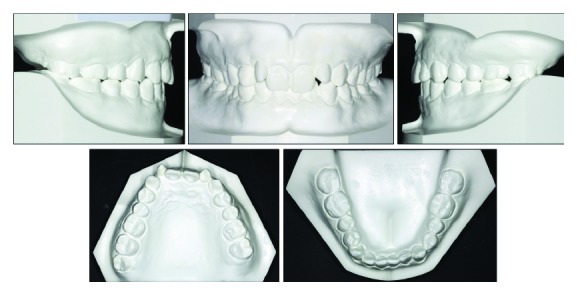
Pretreatment dental casts.

**Figure 3:**
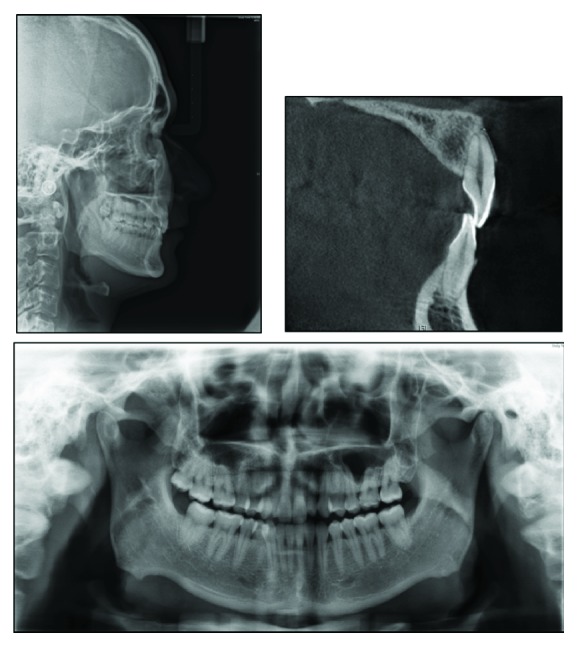
Pretreatment cephalography, panoramic radiograph, and pretreatment image of maxillary left central incisor from CBCT.

**Figure 4:**
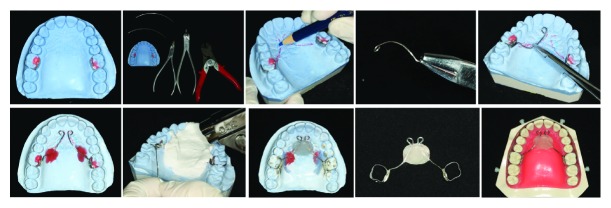
Fabrication of the MNA.

**Figure 5:**
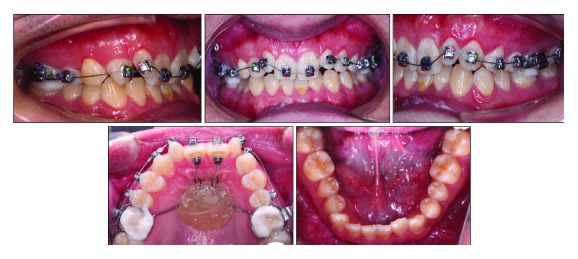
Self-ligating brackets (High Torque) were bonded in the maxillary arch.

**Figure 6:**
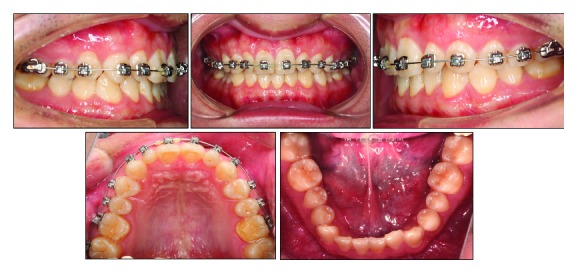
Four months after initial bonding. The MNA and occlusal stop were removed, and the upper arch form was changed from square to ovoid.

**Figure 7:**
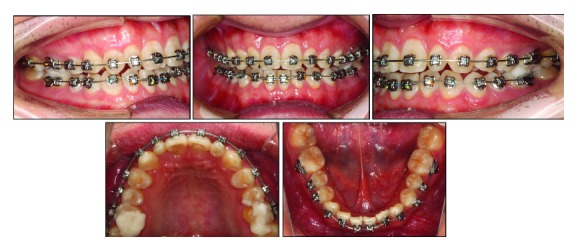
Initial bonding on the lower arch.

**Figure 8:**
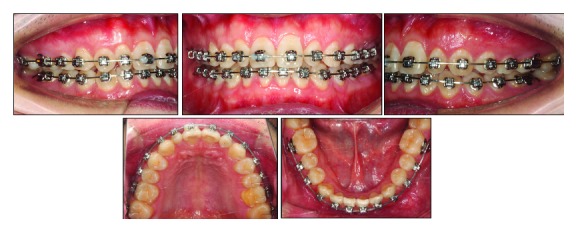
Eighteen months after initial bonding. 0.018 × −0.025-inch nickel-titanium archwire in the maxillary arch and 0.017 × 0.025-inch stainless steel archwire in the mandibular arch were applied.

**Figure 9:**
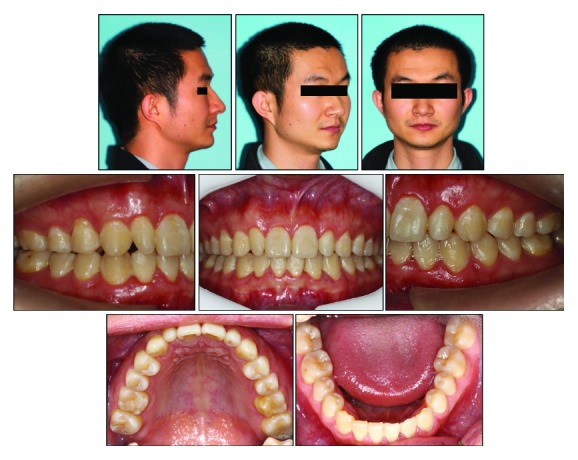
Posttreatment facial and intraoral photographs.

**Figure 10:**
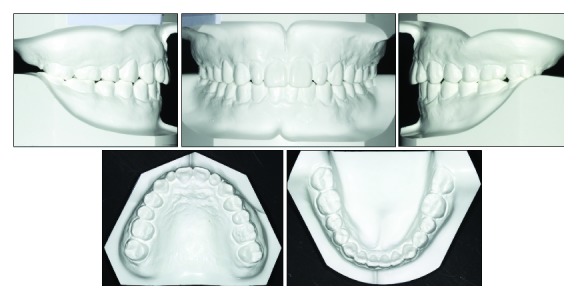
Posttreatment dental casts.

**Figure 11:**
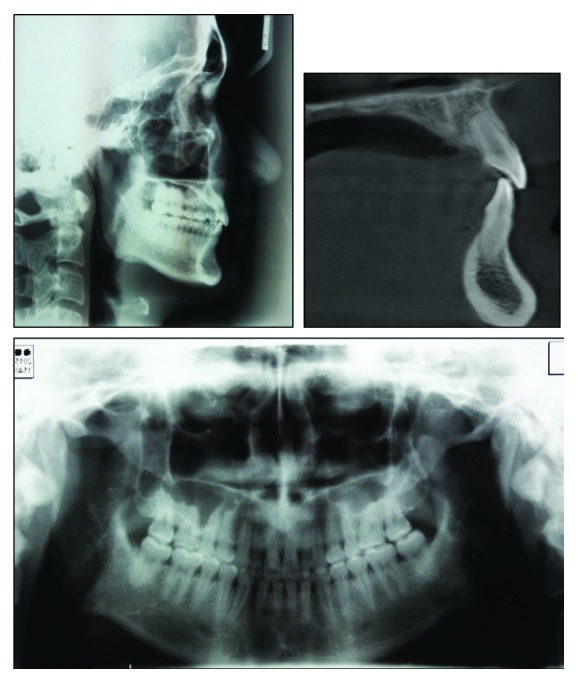
Posttreatment cephalograph, panoramic radiograph, and posttreatment image of maxillary left central incisor from CBCT.

**Figure 12:**
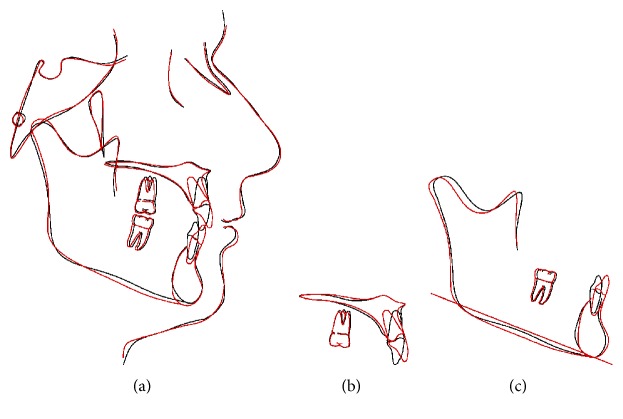
Cephalometric superimpositions showed marked differences between pretreatment (black) and posttreatment (red): (a) SN plane; (b) maxillary plane; (c) mandibular plane.

**Table 1:** Skeletal and dental changes indicated by the cephalometric measurements.

Variables	Norm		Pretreatment	Posttreatment	Difference
	Mean	SD			
Angular (°)					
SNA	82.3	3.5	84.2	81.7	−2.5
SNB	77.6	2.9	79	79.2	0.2
ANB	4.7	1.4	5.2	2.5	−2.7
U1-SN	104.8	5.3	76.8	102.4	25.6
U1/L1	122	6	172.7	141	−31.7
FMA	31.8	4.4	23.7	25.9	2.2
L1/MP	94.7	5.2	79.7	89.5	9.8

Linear (mm)					
Wits appraisal	−1.4	2.6	−1.4	−2.7	−1.3
N-ANS	49	2.2	51.9	51.9	0
ANS-Me	60.8	4.9	55.5	56.9	1.4
L1-Apo	3.9	1.5	−4	0.2	−3.8
Low lip to E-line	3	1.8	−4.7	−4	0.7
Face height ratio (%)	55.4	1.3	51.7	52.3	1.6
